# Eight new freshwater mussels (Unionidae) from tropical Asia

**DOI:** 10.1038/s41598-019-48528-z

**Published:** 2019-08-19

**Authors:** Ivan N. Bolotov, Ekaterina S. Konopleva, Ilya V. Vikhrev, Manuel Lopes-Lima, Arthur E. Bogan, Zau Lunn, Nyein Chan, Than Win, Olga V. Aksenova, Mikhail Yu Gofarov, Alena A. Tomilova, Alexander V. Kondakov

**Affiliations:** 1 Northern Arctic Federal University, Northern Dvina Emb. 17, 163002 Arkhangelsk, Russian Federation; 2 Federal Center for Integrated Arctic Research, Russian Academy of Sciences, Northern Dvina Emb. 23, 163000 Arkhangelsk, Russian Federation; 3 CIBIO/InBIO – Research Center in Biodiversity and Genetic Resources, University of Porto, Campus Agrário de Vairão, Rua Padre Armando Quintas 7, 4485-661 Vairão, Portugal; 4 CIIMAR/CIMAR – Interdisciplinary Centre of Marine and Environmental Research, University of Porto, Terminal de Cruzeiros do Porto de Leixões, Avenida General Norton de Matos, S/N, 4450-208 Matosinhos, Portugal; 5 SSC/IUCN – Mollusc Specialist Group, Species Survival Commission, International Union for Conservation of Nature, c/o The David Attenborough Building, Pembroke Street, CB2 3QZ Cambridge, United Kingdom; 6 North Carolina State Museum of Natural Sciences, 11 West Jones St., Raleigh, NC 27601 United States of America; 7 Fauna & Flora International – Myanmar Program, Yangon, Myanmar; 8 Department of Zoology, Hpa-An University, Hpa-An, Kayin State Myanmar

**Keywords:** Zoology, Biogeography, Taxonomy

## Abstract

Freshwater mussels are sensitive to habitat and water quality, revealing the fastest rates of human-mediated global extinction among aquatic animals. These animals are especially diverse in tropical Asia, the faunas of which are characterized by high levels of endemism. Here we describe four new species and four new subspecies of freshwater mussels from Myanmar. *Leoparreysia whitteni* **sp. nov**., the smallest representative of this genus, was discovered from the Ayeyarwady and Chindwin rivers. *Radiatula myitthanensis* **sp. nov**. and *R. chindwinensis* **sp. nov**. were recorded from the Chindwin Basin, and *R. mouhoti haungthayawensis* **ssp. nov**. has been discovered from the Haungthayaw River. *Indochinella pugio* has been revised with a description of three subspecies: *I. pugio viridissima* **ssp. nov**. from the Sittaung, Bilin and Bago rivers, *I. pugio daweiensis* **ssp. nov**. from the Dawei River, and *I. pugio paradoxa* **ssp. nov**. from the Haungthayaw River. *Yaukthwa elongatula* **sp. nov**., a peculiar species, conchologically resembling representatives of the genus *Solenaia* (Gonideinae) with ultra-elongated shell was found in the Chindwin Basin. Our records highlight that tropical Asia harbors numerous, but still overlooked local endemic lineages of freshwater bivalves, which may be on the brink of extinction due to the high anthropogenic and climate change impacts.

## Introduction

Freshwater bivalves contribute to or provide a plethora of ecosystem functions and services^1^. Currently, freshwater bivalves are among the most threatened groups in the world with 40% of the species being near threatened, threatened or extinct^2^. The interior basin of the USA, Central America, Yangtze Basin, India and Southeast Asia are the most species-rich hotspots of freshwater bivalves at the global scale^2–4^.

Myanmar differs from other tropical Asian countries by a spectacular freshwater fauna with numerous local endemic taxa at the genus and species levels^5–10^. Biogeographically, most of country’s river basins, e.g. the Ayeyarwady, Bago, Sittaung, and Salween, belongs to the Western Indochina Subregion^7^. However, northwestern drainages of the Rakhine Coast, e.g. the Kaladan and Lemro rivers, seem to belong to the Indian Subregion, although the mussel fauna of those basins is still to be explored in detail^11^. Finally, the most eastern edge of the Shan State belongs to the Mekong River basin, and, hence, to the Sundaland Subregion^7^.

Bolotov *et al*.^6^ published the first integrative revision of the freshwater mussel fauna of Myanmar, with a description of two new genera, seven new species and one new subspecies. Two additional new endemic genera, *Indochinella* and *Yaukthwa*, have recently been introduced^7,9^. However, our current knowledge of freshwater mussels of Myanmar is far from being complete, with many lineages still waiting to be described. Further taxonomic research is necessary to develop a national conservation action plan for freshwater bivalves for Myanmar. This plan is urgently needed due to the current high rates of economic development and cropland expansion, leading to the rapid degradation of freshwater habitats^12^.

This study aims to describe four new species and four new subspecies of freshwater mussels from Myanmar. We introduce the new taxa on the basis of an integrative approach combining morphological, molecular and biogeographic evidences. This approach seems to be the most appropriate tool to uncover the diversity of freshwater mollusks in species-rich areas such as the Oriental Region^6–9,13,14^.

## Results

### Phylogeny

**Figure 1:**
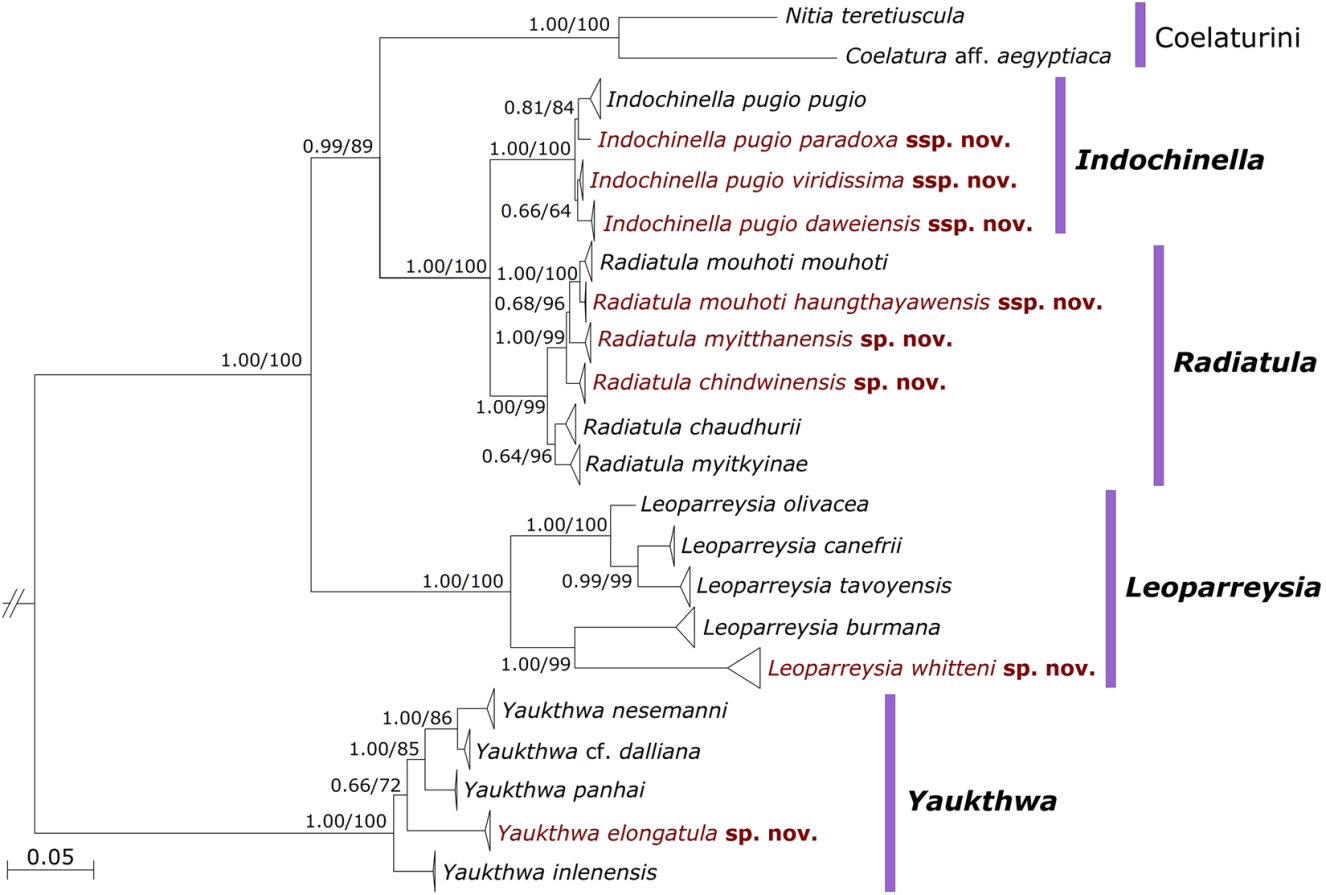
Bayesian phylogeny of the complete data set of mitochondrial and nuclear sequences (five partitions: three codons of *COI* + *16S rRNA* + *28S rRNA*) of the Unionidae genera under discussion: *Leoparreysia*, *Radiatula*, *Indochinella*, and *Yaukthwa*. Scale bar indicates the branch lengths. Black numbers near nodes are Bayesian posterior probabilities (BPP) of MrBayes v. 3.2.6/Ultrafast bootstrap support (BS) values of IQ-TREE. The names of novel taxa are in red. Outgroup is not shown.

**Figure 2:**
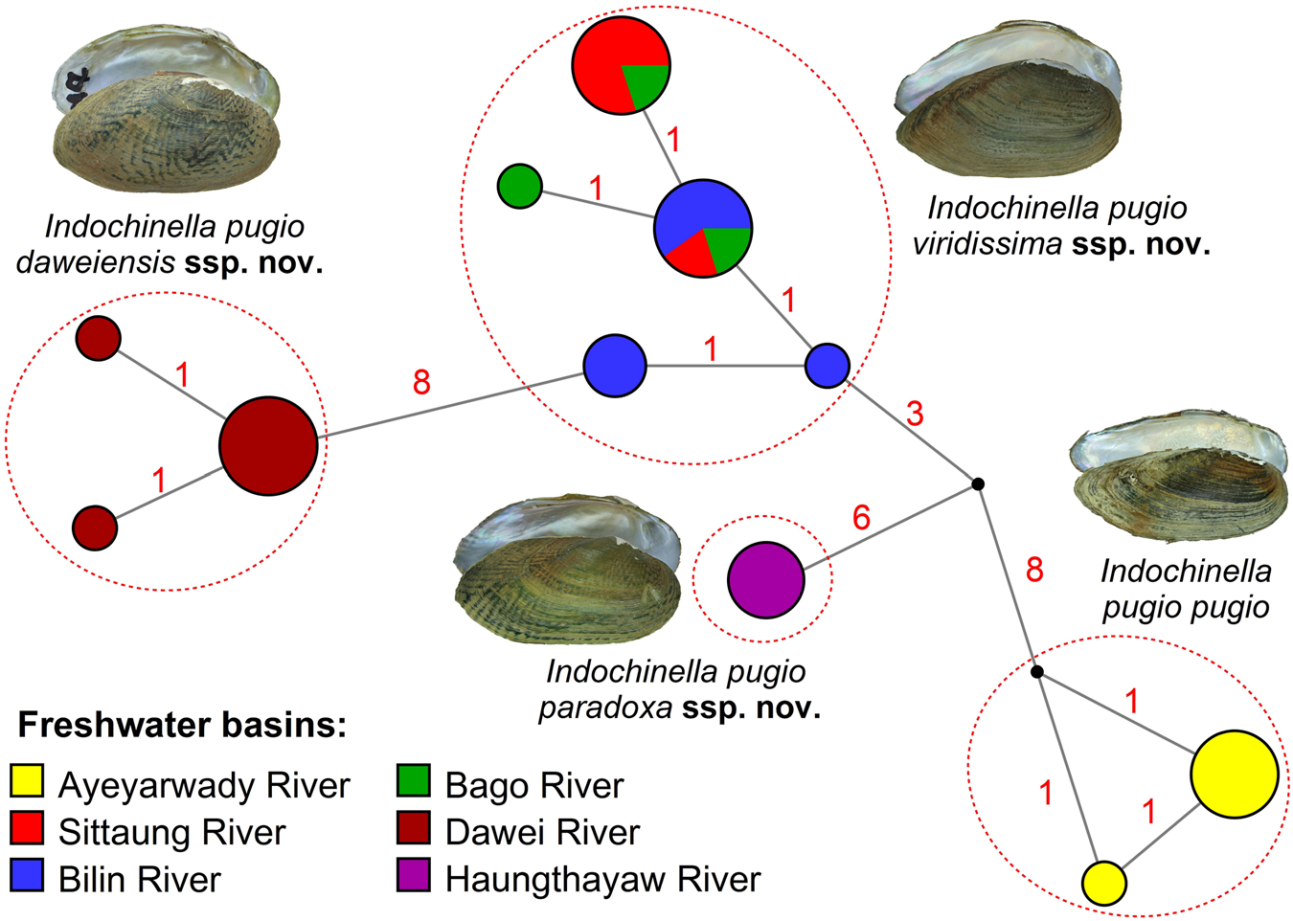
Median-joining haplotype network of the *Indochinella* subspecies based on the *COI* sequences (*N* = 29). The circle size is proportional to the number of available sequences belonging to a certain haplotype (smallest circle = one sequence). The small black dots represent hypothetical ancestral haplotypes. Red numbers near branches indicate the number of nucleotide substitutions between haplotypes. (Photos: Ekaterina S. Konopleva).

Searching with IQ-TREE and MrBayes based on a multi-locus molecular data set with the mitochondrial *cytochrome c oxidase subunit I* (*COI*), *small ribosomal RNA* (*16S rRNA*), and the nuclear *large ribosomal RNA* (*28S rRNA*) gene fragments returned well-resolved phylogenetic models of similar topology (Fig. 1). The four genera under discussion, i.e. *Leoparreysia*, *Indochinella, Radiatula*, and *Yaukthwa*, were recorded as robust, fully supported clades (BS ≥ 99%; BPP = 1.00). To check the relationship between closely related taxa within the genus *Indochinella*, we calculated a median-joining network that shows four distant haplogroups divided by corresponding river basins (Fig. 2).

### Species delimitation

**Figure 3:**
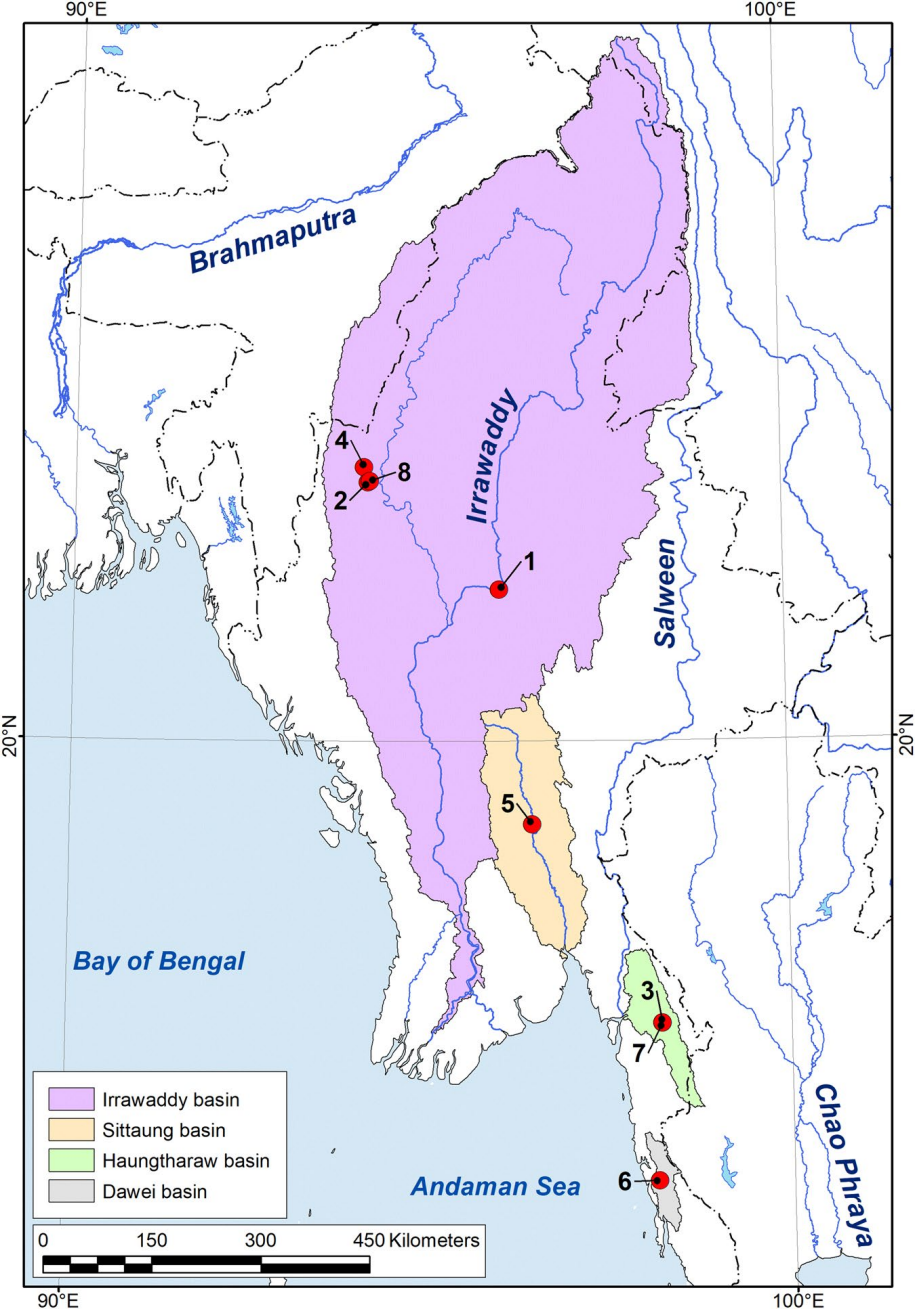
Map of the type localities of new freshwater mussel taxa from Myanmar. (1) *Leoparreysia whitteni* **sp. nov**.: Ayeyarwady River, Su Taung Seik area, Sagaing, Mandalay. (2) *Radiatula myitthanensis* **sp. nov**.: Myit Tha (Manipur) River. (3) *R. mouhoti haungthayawensis* **ssp. nov**.: Haungthayaw River upstream of Kawkareik town. (4) *R. chindwinensis* **sp. nov**.: tributary of Nay Chin Sayar River, Chindwin Basin. (5) *Indochinella pugio viridissima* **ssp. nov**.: Myit Kyi Pauk Stream, Sittaung Basin. (6) *I. pugio daweiensis* **ssp. nov**.: Dawei River. (7) *I. pugio paradoxa* **ssp. nov**.: Haungthayaw River upstream of Kawkareik town. (8) *Yaukthwa elongatula* **sp. nov**.: Chindwin River. The map was developed using ESRI ArcGIS 10 software (www.esri.com/arcgis). The topographic base of the map was compiled with Natural Earth Free Vector and Raster Map Data (www.naturalearthdata.com), GSHHG version 2.3.7 (www.soest.hawaii.edu/pwessel/gshhg)^35^, and the HydroSHEDS database (www.hydrosheds.org)^36,37^. (Map: Mikhail Yu. Gofarov).

**Figure 4:**
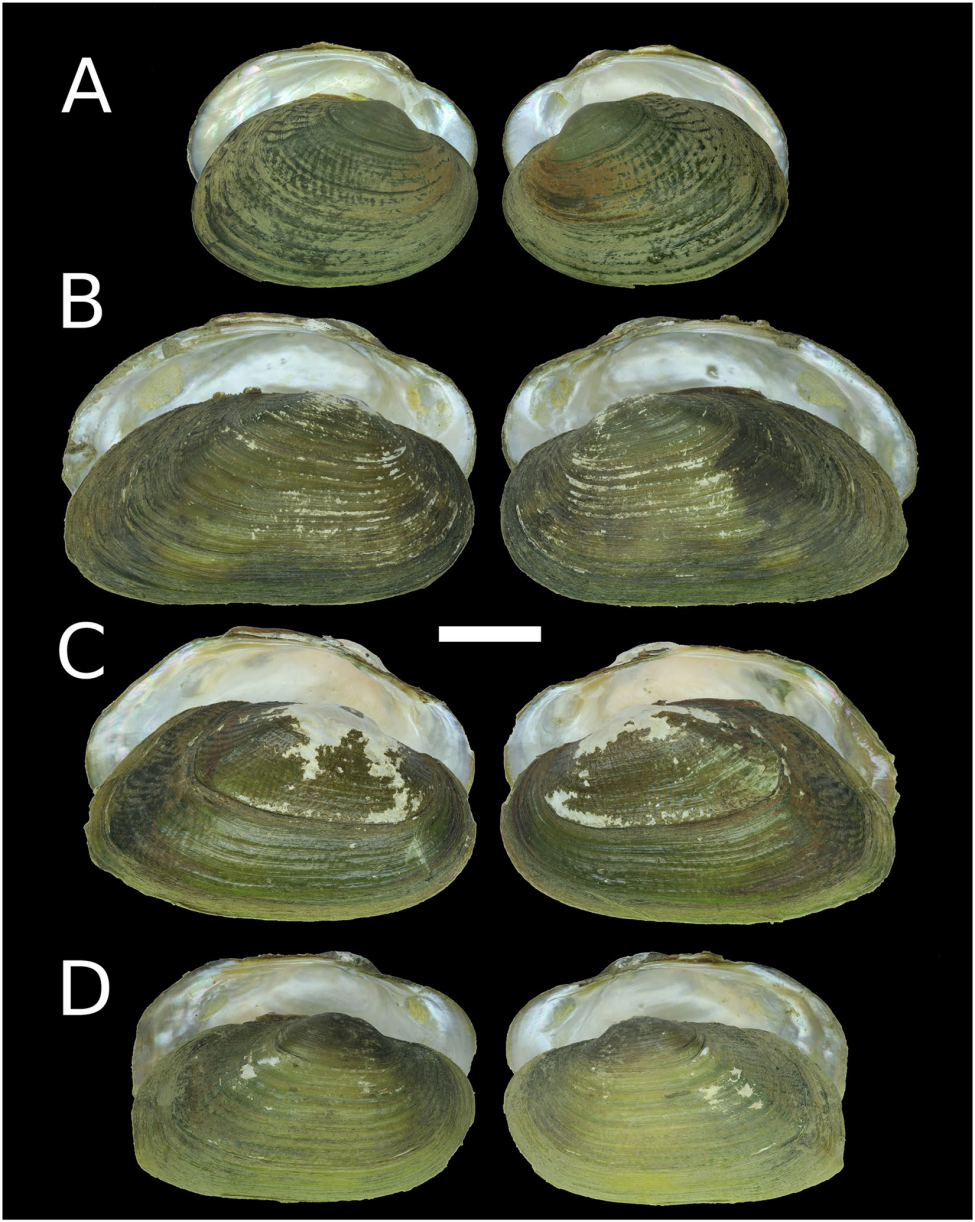
Shells of new freshwater mussel taxa from Myanmar. (**A**) *Leoparreysia whitteni* **sp. nov**. [holotype RMBH biv 449]. (**B**) *Radiatula myitthanensis* **sp. nov**. [holotype RMBH biv 337_3]. (**C**) *R. mouhoti haungthayawensis* **ssp. nov**. [holotype RMBH biv 360_1]. (**D**) *R. chindwinensis* **sp. nov**. [holotype RMBH biv357_3]. (Photos: Ekaterina S. Konopleva).

**Figure 5:**
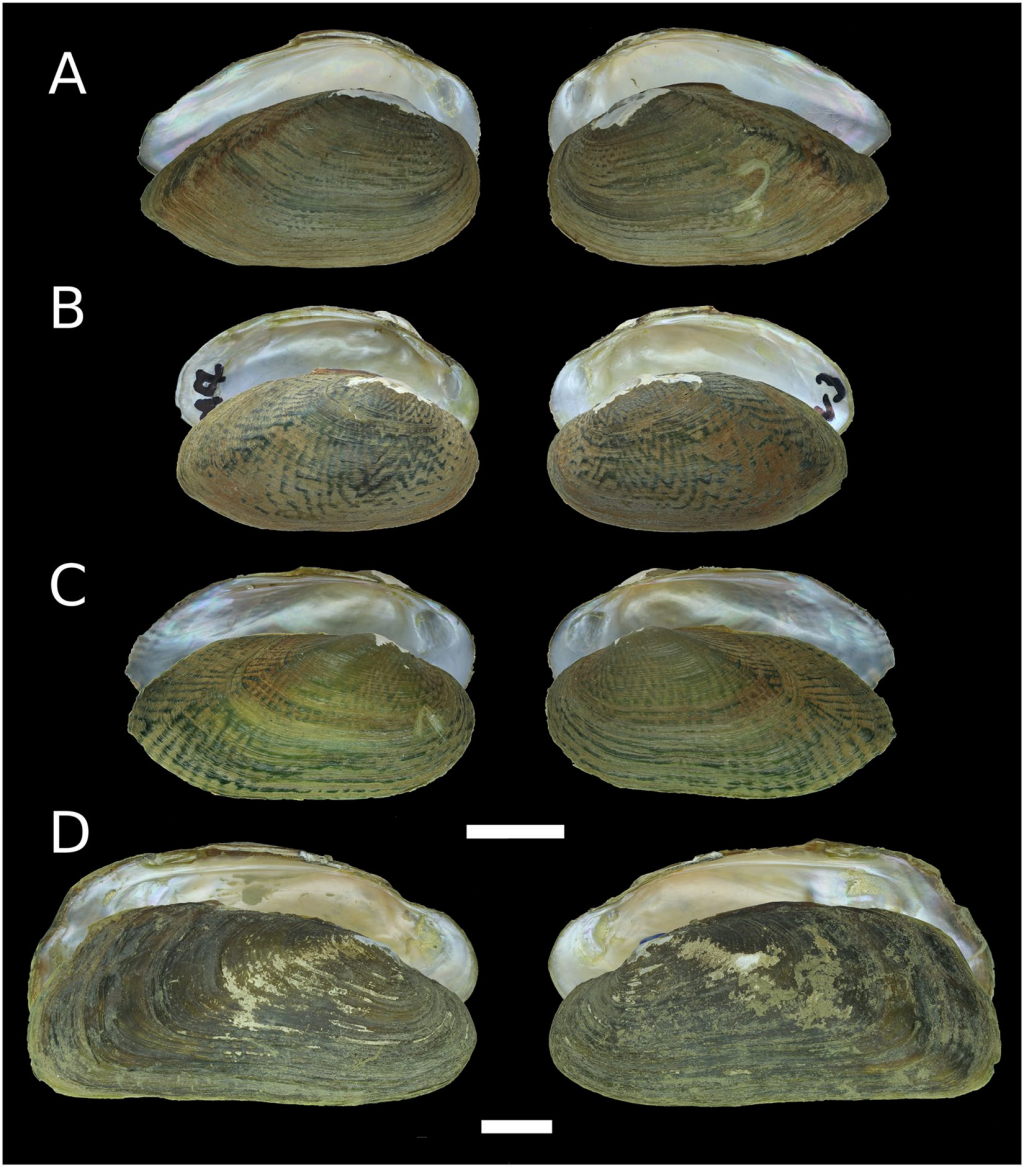
Shells of new freshwater mussel taxa from Myanmar. (**A**) *Indochinella pugio viridissima* **ssp. nov**. [holotype RMBH biv 251_3]. (**B**) *I. pugio daweiensis* **ssp. nov**. [holotype RMBH biv 147_3]. (**C**) *I. pugio paradoxa* **ssp. nov**. [holotype RMBH biv 361_1]. (**D**) *Yaukthwa elongatula* **sp. nov**. [paratype RMBH biv 341_2]. (Photos: Ekaterina S. Konopleva).

**Figure 6:**
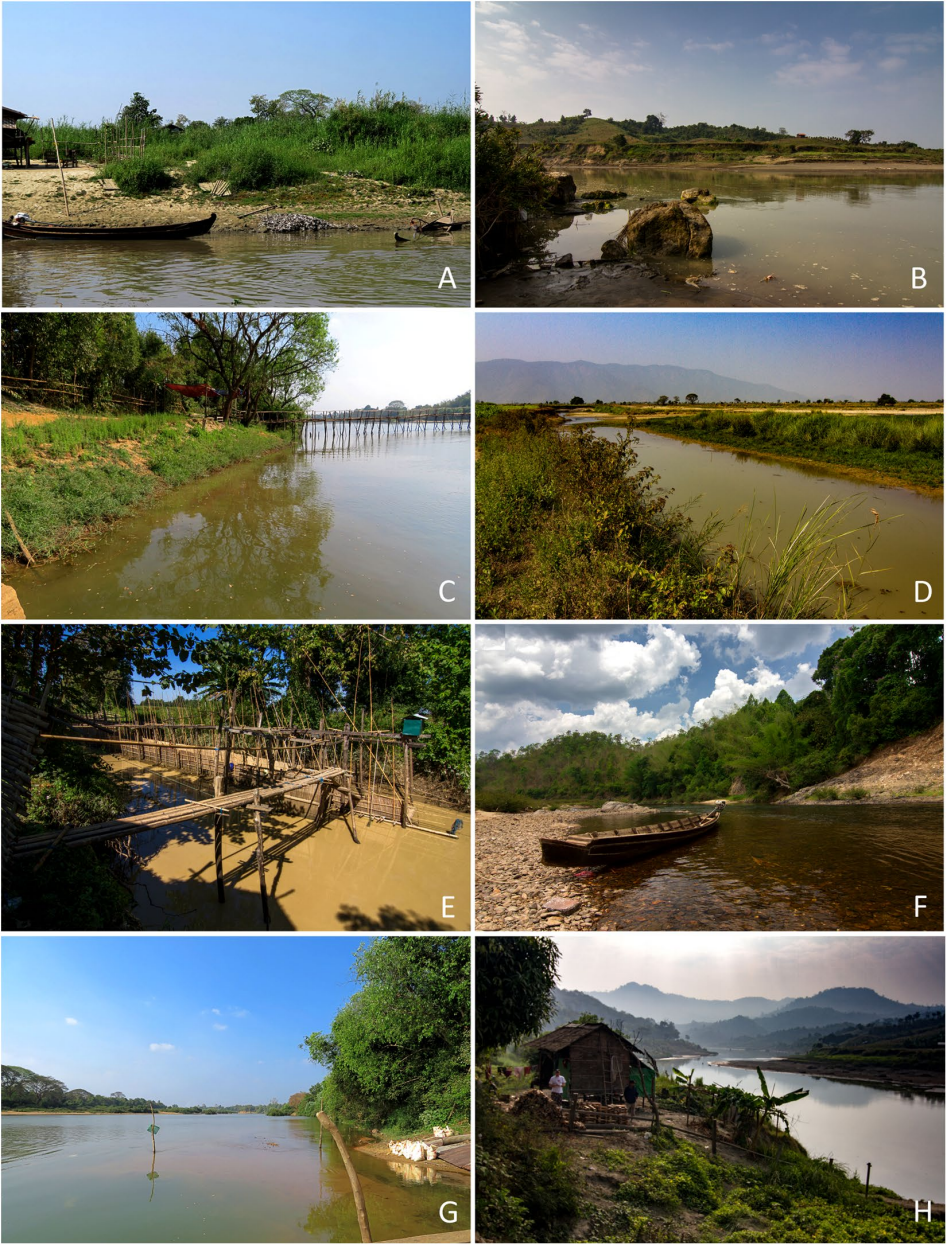
Type localities and habitats of new freshwater mussel taxa from Myanmar. (**A**) *Leoparreysia whitteni* **sp. nov**.: Ayeyarwady River, Su Taung Seik area, Sagaing, Mandalay [type locality]. (**B**) *Radiatula myitthanensis* **sp. nov**.: Myit Tha (Manipur) River [type locality]. (**C**) *R. mouhoti haungthayawensis* **ssp. nov**.: Haungthayaw River upstream of Kawkareik town [type locality]. (**D**) *R. chindwinensis* **sp. nov**.: tributary of Nay Chin Sayar River, Chindwin Basin [type locality]. (**E**) *Indochinella pugio viridissima* **ssp. nov**.: Myit Kyi Pauk Stream, Sittaung Basin [type locality]. (**F**) *I. pugio daweiensis* **ssp. nov**.: Dawei River [type locality]. (**G**) *I. pugio paradoxa* **ssp. nov**.: Haungthayaw River upstream of Kawkareik town [type locality]. (**H**) *Yaukthwa elongatula* **sp. nov**.: Myit Tha (Manipur) River [habitat]. (Photos: Nyein Chan [A,C,G] and Ilya V. Vikhrev [B,D–F,H]).

The species delimitation analysis through the BEAST2 package STACEY with an initial grouping of taxa supported our hypothesis on 20 putative species-level lineages (probability = 0.80). Eight of these taxa are new to science and are described here (Tables 1–2; Figs 1–6). Each new taxon can be clearly distinguished from sister species or subspecies by molecular diagnosis (Table 2). Four lineages are assigned to valid species, i.e. *Leoparreysia whitteni* **sp. nov**., *Radiatula myitthanensis* **sp. nov**., *R. chindwinensis* **sp. nov**., and *Yaukthwa elongatula* **sp. nov**., because they share high levels of molecular divergence from sister clades (mean uncorrected *COI p*-distance = 3.2–9.3%). In contrast, four novel lineages sharing rather low molecular divergence from nearest neighbors (mean uncorrected *COI p*-distance = 1.4–1.6%) are introduced as the following subspecies: *Indochinella pugio viridissima* **ssp. nov**., *I. pugio daweiensis* **ssp. nov**., *I. pugio paradoxa* **ssp. nov**., and *Radiatula mouhoti haungthayawensis* **ssp. nov**. However, *Indochinella pugio daweiensis* **ssp. nov**. and *I. pugio paradoxa* **ssp. nov**. have one or two diagnostic nucleotide substitutions in the nuclear *28S rRNA* gene fragment (Table 2) that usually indicates species-level differences between the Unionidae taxa.

**Table 1:** Shell measurements and reference DNA sequences for the type series of new freshwater mussel taxa (Unionidae) from Myanmar.

Taxon	Status of Specimen	Specimen Voucher*	Shell Length, mm	Shell Height, mm	Shell Width, mm	NCBI’s GenBank acc. nos.		
						*COI*	*16S rRNA*	*28S rRNA*
*Leoparreysia whitteni* **sp. nov**.	Holotype	biv449	28.7	19.5	12.2	MK372449	MK372476	MK372508
	Paratype	biv349	27.9	17.6	10.3	MK372411	MK372459	MK372489
	Paratype	biv435	26.7	18.0	12.4	MK372446	MK372475	MK372507
*Radiatula myitthanensis* **sp. nov**.	Holotype	biv337_3	41.0	21.7	15.5	MK372396	MK372450	MK372477
	Paratype	biv348_1	42.4	22.1	15.5	MK372409	MK372457	MK372487
	Paratype	biv337_4	42.0	22.2	17.2	n/a	n/a	n/a
	Paratype	biv337_8	35.5	19.4	13.8	n/a	n/a	n/a
	Paratype	biv337_11	39.6	21.7	15.0	n/a	n/a	n/a
	Paratype	biv337_17	42.9	22.4	16.0	n/a	n/a	n/a
	Paratype	biv337_13	45.1	23.7	16.6	n/a	n/a	n/a
	Paratype	biv337_20	38.6	21.3	14.5	n/a	n/a	n/a
	Paratype	biv337_10	39.1	21.4	14.2	n/a	n/a	n/a
	Paratype	biv337_12	37.6	21.7	14.8	n/a	n/a	n/a
	Paratype	biv337_15	41.7	23.1	17.0	n/a	n/a	n/a
*Radiatula mouhoti haungthayawensis* **ssp. nov**.	Holotype	biv360_1	39.4	22.5	17.8	MK372417	MK372463	MK372493
	Paratype	biv360_2	33.7	18.8	14.0	MK372418	MK372464	MK372494
	Paratype	biv360_3	31.3	18.0	14.2	MK372419	n/a	MK372495
	Paratype	biv360_4	31.5	17.6	14.4	n/a	n/a	n/a
	Paratype	biv360_5	30.2	17.4	12.5	n/a	n/a	n/a
	Paratype	biv360_7	25.3	15.2	11.1	n/a	n/a	n/a
	Paratype	biv360_8	26.4	15.3	10.7	n/a	n/a	n/a
*Radiatula chindwinensis* **sp. nov**.	Holotype	biv357_3	34.3	18.5	13.5	MK372416	n/a	n/a
	Paratype	biv345_2	29.5	16.5	10.2	MK372405	MK372453	MK372483
	Paratype	biv348_2	34.5	19.5	13.8	MK372410	MK372458	MK372488
	Paratype	biv345_1	32.1	17.8	11.8	MK372404	MK372452	MK372482
	Paratype	biv357_2	33.8	18.6	15.0	MK372415	n/a	n/a
	Paratype	biv357_1	34.4	19.3	13.3	MK372414	MK372462	MK372492
	Paratype	biv357_4	28.7	16.7	13.1	n/a	n/a	n/a
*Indochinella pugio viridissima* **ssp. nov**.	Holotype	biv_251_3	34.8	18.6	14.1	MF352244	MF352314	MF352372
	Paratype	biv_251_1	32.3	16.3	11.6	MF352242	MF352312	MF352370
	Paratype	biv_251_2	32.7	17.0	11.9	MF352243	MF352313	MF352371
	Paratype	biv371_1	44.3	21.3	16.1	MK372426	MK372468	MK372500
	Paratype	biv371_2	42.4	20.6	15.8	MK372427	MK372469	MK372501
	Paratype	biv371_3	44.8	21.2	14.9	MK372428	n/a	n/a
	Paratype	biv375_1	34.8	18.0	12.5	MK372429	n/a	n/a
	Paratype	biv375_2	32.2	16.3	11.5	MK372430	n/a	n/a
	Paratype	biv375_3	33.0	16.8	12.2	MK372431	n/a	n/a
	Paratype	biv377_2	35.4	17.9	13.0	MK372432	n/a	n/a
	Paratype	biv377_3	37.4	19.3	13.5	MK372433	n/a	n/a
	Paratype	biv381_3	45.8	23.8	16.9	MK372434	n/a	n/a
	Paratype	biv381_4	36.4	18.7	12.8	MK372435	n/a	n/a
	Paratype	biv386_2	37.0	20.1	14.1	MK372436	n/a	n/a
*Indochinella pugio daweiensis* **ssp. nov**.	Holotype	biv_147_3	31.0	16.6	12.6	KX865852	KX865623	KX865724
	Paratype	biv_147_10	27.9	14.8	12.2	KX865853	KX865624	KX865725
	Paratype	biv_147_18	26.1	13.6	9.9	KX865854	KX865625	KX865726
	Paratype	biv_148_4	32.3	17.5	14.2	KX865855	KX865626	KX865727
	Paratype	biv_148_7	32.6	17.5	14.8	KX865856	KX865627	KX865728
	Paratype	biv_148_15	37.4	19.6	15.7	KX865857	KX865628	KX865729
	Paratype	biv_147_30	30.4	16.4	12.2	MK372395	n/a	n/a
	Paratype	biv_147_1	32.0	16.6	13.3	n/a	n/a	n/a
	Paratype	biv_147_2	30.3	16.3	11.9	n/a	n/a	n/a
	Paratype	biv_147_4	31.8	17.4	12.0	n/a	n/a	n/a
	Paratype	biv_148_2	36.9	20.4	14.9	n/a	n/a	n/a
	Paratype	biv_148_3	38.0	20.0	15.5	n/a	n/a	n/a
*Indochinella pugio paradoxa* **ssp. nov**.	Holotype	biv361_1	36.1	17.3	12.5	MK372420	MK372465	MK372496
	Paratype	biv361_2	35.6	18.0	13.1	MK372421	MK372466	MK372497
	Paratype	biv361_3	28.6	17.7	10.6	MK372422	MK372467	MK372498
	Paratype	biv361_4	29.0	16.4	10.8	n/a	n/a	n/a
	Paratype	biv361_5	27.1	15.2	10.4	n/a	n/a	n/a
	Paratype	biv361_6	26.6	14.8	9.9	n/a	n/a	n/a
	Paratype	biv361_7	28.2	15.1	10.0	n/a	n/a	n/a
	Paratype	biv361_8	23.3	13.1	8.2	n/a	n/a	n/a
	Paratype	biv361_9	24.2	12.3	8.3	n/a	n/a	n/a
*Yaukthwa elongatula* **sp. nov**.	Paratype	biv341_2	64.3	28.1	13.2	MK372400	MK372451	MK372480
	Paratype	biv346_3	51.3	24.2	12.4	MK372408	MK372456	MK372486
	Paratype	biv346_1	55.6	25.3	12.6	MK372406	MK372454	MK372484
	Paratype	biv344_1	58.6	26.8	14.9	MK372402	n/a	n/a
	Paratype	biv344_2	52.2	23.5	12.0	n/a	n/a	n/a
	Paratype	biv344_3	35.1	16.4	8.2	MK372401	n/a	MK372481
	Holotype	biv346_2	48.2	23.7	12.1	MK372406	MK372454	MK372484
	Paratype	biv341_3	49.2	21.3	12.5	MK372401	n/a	MK372481
	Paratype	biv339_1	59.4	26.3	14.8	MK372397	n/a	MK372478
	Paratype	biv339_2	57.0	23.2	14.3	MK372398	n/a	MK372479
	Paratype	biv339_3	58.7	25.1	14.3	MK372399	n/a	n/a
	Paratype	biv339_7	51.2	24.7	13.1	n/a	n/a	n/a

*Type series are deposited in the RMBH – Russian Museum of Biodiversity Hotspots, Federal Center for Integrated Arctic Research, Russian Academy of Sciences, Arkhangelsk, Russia. n/a – not available.

**Table 2:** Molecular diagnoses of the new freshwater mussel taxa (Unionidae) from Myanmar.

Taxon	Mean *COI p*-distance from the nearest neighbor, %	The nearest neighbor of new taxon	Fixed nucleotide differences based on the sequence alignment of congeners		
			*COI*	*16S rRNA*	*28S rRNA*
*Leoparreysia whitteni* **sp. nov**.	9.29	*L. olivacea*	15 C, 56 C, 62 G, 74 A, 77 G, 92 A, 101 G, 128 C, 182 G, 227 C, 296 A, 308 G, 323 G, 353 G, 356 T, 407 C, 458 G, 524 A, 539 G, 554 G, 656 A	12 C, 18 T, 20 C, 49 A, 51 T, 110 T, 144 C, 200 C, 233 C, 234 T, 238 G, 241 C, 248 A, 250 A, 264 C, 300 C, 324 C, 329 C	127 С, 413 T, 432 T/Y, 454 T, 469 C, 470 G, 471 A, 484 G, 494 T, 523 G, 524 A, 531 T, 542 C, 550 C, 559 G, 567 G, 568 A, 584 T, 601 G, 621 G, 622 T, 623 T, 631 T, 647 T, 654 G, 655 T, 682 T, 686 A, 702 T, 709 A, 710 A, 747 C, 752 T, 760 C, 758 A
*Radiatula myitthanensis* **sp. nov**.	3.21	*R. mouhoti*	389 A, 422 G, 425 G, 434 A, 443 C, 527 C, 578 A	240 G, 317 C, 331 A	None
*R. mouhoti haungthayawensis* **ssp. nov**.	1.36	*R. mouhoti*	35 C, 254 G	None	None
*R. chindwinensis* **sp. nov**.	3.33	*R. mouhoti*	122 A, 212 A, 293 G, 308 A, 364 G, 449 C, 479 A	53 G	None
*Indochinella pugio viridissima* **ssp. nov**.	1.61	*I. pugio paradoxa* **ssp. nov**.	57 C	None	None
*I. pugio daweiensis* **ssp. nov**.	1.59	*I. pugio viridissima* **ssp. nov**.	86 G, 110 G, 347 A, 402 C, 557 C, 635 C, 644 A, 659 C	17 A, 314 C, 316 T, 324 T	759 A
*I. pugio paradoxa* **ssp. nov**.	1.61	*I. pugio viridissima* **ssp. nov**.	8 G, 291 C, 341 T, 404 G, 443 C, 608 G	328 G	507 C, 771 G
*Yaukthwa elongatula* **sp. nov**.	8.28	*Y. inlenensis*	6 C, 15 C, 17 A, 35 T, 50 C, 56 C, 71 G, 95 A, 167 A, 182 C, 194 C, 206 T, 242 G, 248 C, 275 A, 296 A, 302 T, 319 C, 338 C, 365 C, 389 A, 425 A, 443 C, 506 C, 539 G, 542 A, 587 G, 629 A, 638 T	154 A, 155 C, 238 G, 247 G, 249 T, 253 A, 270 G, 336 C, 342 T, 469 C	210 G, 212 T, 496 A, 589 Del, 608 G

Del = deletion mutation.

### Taxonomic account

Family Unionidae Rafinesque, 1820

**Subfamily Parreysiinae Henderson, 1935**

Type genus: *Parreysia* Conrad, 1853 (by original designation)

**Tribe Leoparreysiini Vikhrev, Bolotov & Kondakov, 2017**

Type genus: *Leoparreysia* Vikhrev, Bolotov & Aksenova, 2017 (by original designation)

**Genus** ***Leoparreysia*** **Vikhrev, Bolotov & Aksenova, 2017**

Type species: *Leoparreysia canefrii* Vikhrev, Bolotov & Kondakov, 2017 (by original designation)

***Leoparreysia whitteni*** **sp. nov**.

Figures 3, 4A, 6A, Tables 1–2.

Type locality: Myanmar: Ayeyarwady River, Su Taung Seik area, Sagaing, Mandalay [21.8893°N, 95.9978°E].

Holotype: RMBH biv_449: Myanmar: Ayeyarwady River, Su Taung Seik area, Sagaing, Mandalay, 21.8893°N, 95.9978°E, 4.iii.2018, Bolotov, Vikhrev, Nyein Chan and local villagers leg.

Paratypes: Myanmar: Chindwin River, 23.1918°N, 94.3217°E, 2.ii.2018, 1 specimen (RMBH biv_349); Myanmar: Ayeyarwady River, Mandalay, 21.9574°N, 96.0510°E, 3.iii.2018, 1 specimen (RMBH biv_435), Bolotov, Vikhrev, Nyein Chan and local villagers leg.

Etymology: This species is named in memory of the late Dr. Tony Whitten (1953–2017), a famous conservation biologist, Fauna & Flora International – Asia Pacific, United Kingdom.

Diagnosis: The new species is morphologically most similar to *Leoparreysia burmana* (Blanford, 1869) but can be distinguished from it by a more elongated shell, and different location and size of umbo (small subinequilateral umbo in the new species vs. large strongly inequilateral umbo in *L. burmana*). *Leoparreysia whitteni* **sp. nov**. has a high genetic divergence from all other taxa in this genus by the *COI*, *16S rRNA* and *28S rRNA* gene fragments (Table 2).

Description: Very small mussel. Shell length 26.7–28.7 mm, height 17.6–19.5 mm, width 10.3–12.4 mm. Shell ovate, subinequilateral, slightly inflated and thick. Posterior margin broader than anterior margin, with arched bars along the slope. Strong v-shaped sculpture all over the shell surface (except for one specimen), but it is slightly visible or absent on umbo area. Periostracum yellow-green or brown-green, nacre whitish, somewhat shining. Pseudocardinal teeth strong and very indented, by two teeth in each valve, of typical *Leoparreysia* shape. Lateral teeth moderately short, curved, two teeth in left valve, one tooth in right valve. Anterior muscle attachment scar rounded, more or less deep. Posterior muscle attachment scar shallow.

Habitat and ecology: Silty-clay sites in large rivers (Fig. 6A).

Distribution: Ayeyarwady and Chindwin rivers, Ayeyarwady Basin, Myanmar.

Comments: There are only three available specimens of this species, each from a different locality. The specimen from the Chindwin River has smooth periostracum and no shell sculpture, only bars along the posterior margin.

### Tribe Indochinellini Bolotov, Pfeiffer, Vikhrev & Konopleva, 2018

Type genus: *Indochinella* Bolotov, Pfeiffer, Vikhrev & Konopleva, 2018 (by original designation)

**Genus** ***Radiatula*** **Simpson, 1900**

Type species: *Unio crispisulcatus* Benson, 1862 (by original designation)

***Radiatula myitthanensis*** **sp. nov**.

Figures 3, 4B, 6B, Tables 1–2.

Type locality: Myanmar: Myit Tha (Manipur) River [23.2285°N, 94.1434°E].

Holotype: RMBH biv337_3: Myanmar: Myit Tha (Manipur) River, 23.2285°N, 94.1434°E, 2.ii.2018, Bolotov, Vikhrev, Lopes-Lima, Nyein Chan and local villagers leg.

Paratypes: type locality, 2.ii.2018, 9 specimens (RMBH no. biv_337); Chindwin River, 23.1918°N, 94.3217°E, 2.ii.2018, 1 specimen (RMBH biv_348_1), Bolotov, Vikhrev, Lopes-Lima, Nyein Chan and local villagers leg.

Etymology: The name of this species is derived from its type locality, the Myit Tha River.

Diagnosis: This species is morphologically and genetically close to *Radiatula mouhoti* Vikhrev, Bolotov & Konopleva 2017, but differs from it by more elongated and rounded posterior margin, less developed posterior muscle scar and smaller umbo. *Radiatula myitthanensis* **sp. nov**. is also externally similar to *R. chindwinensis* **sp. nov**., but it differs from the latter species by a rounded posterior slope (vs. truncated posterior slope in *R. chindwinensis* **sp. nov**.) and curved ventral margin (vs. straighter ventral margin in *R. chindwinensis* **sp. nov**.). The new species also differs from all the congeners by fixed nucleotide substitutions in the *COI* and *16S rRNA* gene fragments (Table 2).

Description: Shell length 35.5–45.1 mm, height 19.4–23.7 mm, width 13.8–17.2 mm. Shell ovate-elongated, subinequilateral, somewhat inflated and thick. Dorsal margin slightly curved, ventral margin straight. Anterior margin rounded; posterior slope smooth, margin covered by small wrinkles from the beak. The umbo area has poorly visible v-shaped sculpture, corrugated. Periostracum jade-green, concentrically striated; nacre yellow-whitish. Right valve with a single slightly curved lateral tooth and two pseudocardinal teeth, anterior tooth small and lamellar; posterior tooth rectangular and more or less indented. Left valve with two slightly curved lateral teeth and two pseudocardinal teeth, anterior tooth rectangular and ribbed, posterior tooth smaller and pyramidal. Anterior muscle attachment scar ovate, well pronounced. Posterior muscle attachment scar less well marked.

Habitat and ecology: Sites with clay substrate and large stones in large rivers (Fig. 6B).

Distribution: Myit Tha (Manipur) and Chindwin rivers, Ayeyarwady Basin, northwestern Myanmar.

***Radiatula mouhoti haungthayawensis*** **ssp. nov**.

Figures 3, 4C, 6C, Tables 1–2.

Type locality: Myanmar: Haungthayaw River upstream of Kawkareik town [16.4714°N, 98.2182°E].

Holotype: RMBH biv360_1: Myanmar: Haungthayaw River upstream of Kawkareik town, 16.4714°N, 98.2183°E, 9.ii.2018, Nyein Chan leg.

Paratypes: type locality, 9.ii.2018, 6 specimens (RMBH nos. biv360_2, biv360_3, biv360_4, biv360_5, biv360_7, and biv360_8), Nyein Chan leg.

Etymology: The name of this subspecies refers to the Haungthayaw River, its type locality.

Diagnosis: The new subspecies is genetically close to *Radiatula mouhoti* Vikhrev, Bolotov & Konopleva, 2017, but is morphologically more similar to *R. chaudhurii* (Preston, 1912). *Radiatula mouhoti haungthayawensis* differs from the latter species by possessing a smoother periostracum, a stronger inflation, and a curved ventral margin. The new taxon also differs from the nominative subspecies by two diagnostic nucleotide substitutions in the *COI* gene fragment (Table 2).

Description: Small mussel. Shell length 26.4–39.4 mm, height 15.2–22.5 mm, width 10.7–17.8 mm. Shell elongated, thin, subinequilateral. Anterior end rounded, ventral and dorsal margin curved; posterior side truncated, covered by small wrinkles. The umbo corrugated, with w-shaped sculpture, continued along all over the shell surface. Periostracum olive-green with pinkish patches, concentrically striated; nacre blue-whitish with yellow umbo cavity. Right valve with a single slightly curved lateral tooth and two pseudocardinal teeth, anterior tooth reduced; posterior tooth pyramidal or more rectangular and can be slightly indented. Left valve with two slightly curved lateral teeth and two pseudocardinal teeth, anterior tooth rectangular, posterior tooth smaller and pyramidal. Anterior muscle attachment scar ovate and well-marked. Posterior muscle attachment scar rounded and less pronounced.

Habitat and ecology: This subspecies was collected from a single location, an upstream pool site of the river with sandy-clay substrate (Fig. 6C).

Distribution: Haungthayaw River, southeastern Myanmar.

***Radiatula chindwinensis*** **sp. nov**.

Figures 3, 4D, 6D, Tables 1–2.

Type locality: Myanmar: tributary of Nay Chin Sayar River, Chindwin Basin [23.4160°N, 94.0875°E].

Holotype: RMBH biv 357_3: Myanmar: tributary of Nay Chin Sayar River, Chindwin Basin, 23.4160°N, 94.0875°E, 4.ii.2018, Bolotov, Vikhrev, Lopes-Lima, Nyein Chan and local villagers leg.

Paratypes: type locality, 4.ii.2018, 2 specimens (RMBH nos. biv357_1 and biv357_2); Myanmar: Myit Tha (Manipur) River, 23.2006°N, 94.2214°E, 2.ii.2018, 2 specimens (RMBH nos. biv345_1 and biv345_2); Myanmar: Chindwin River, 23.1918°N, 94.3217°E, 2.ii.2018, 1 specimen (RMBH biv348_2), Bolotov, Vikhrev, Lopes-Lima, Nyein Chan and local villagers leg.

Etymology: The new species is named after the Chindwin River, the largest tributary of the Ayeyarwady Basin, from which the species was collected.

Diagnosis: The new species is conchologically similar to *Radiatula mouhoti* and *R. myitthanensis* **sp. nov**., but can be distinguished from these taxa by more straight dorsal margin and smooth pseudocardinal teeth without marked sculpture (vs. more curved dorsal margin and strongly sculptured pseudocardinal teeth in *R. mouhoti* and *R. myitthanensis* **sp. nov**.). The new species also differs from all the congeners by fixed nucleotide substitutions in the *COI* and *16S rRNA* gene fragments (Table 2).

Description: Small mussel. Shell length 28.7–34.5 mm, height 16.7–19.5 mm, width 10.2–15.0 mm. Shell elongate-ovate, rather thin, nearly equilateral in some specimens, not inflated. Anterior end rounded, slightly shifted upward at some specimens, ventral and dorsal margin straight or slightly curved; posterior side truncated, covered by small arched bars from umbo along the slope. Umbo corrugated, with unpronounced v-shaped sculpture, continued until the middle or ventral margin of the shell. Periostracum olive-green with yellow and dark regions, concentrically striated; nacre blue-whitish. Two lateral teeth on the left valve and a single tooth on the right valve, straight or slightly curved. Right valve with two pseudocardinal teeth, anterior tooth reduced; posterior tooth pyramidal or trapezoidal. Left valve with two pseudocardinal teeth, anterior tooth pyramidal or rectangular, posterior tooth smaller and pyramidal. Anterior muscle attachment scar ovate, rather deep. Posterior muscle attachment scar rounded and shallow.

Habitat and ecology: Clay sites in large rivers (Fig. 6D).

Distribution: Nay Chin Sayar, Myit Tha (Manipur) and Chindwin rivers, Ayeyarwady Basin, northwestern Myanmar.

**Genus** ***Indochinella*** **Bolotov, Pfeiffer, Vikhrev & Konopleva, 2018**

Type species: *Unio pugio* Benson, 1862 (by original designation)

***Indochinella pugio viridissima*** **ssp. nov**.

= *Oxynaia* sp. ‘Taungoo’ sensu Bolotov *et al*. (2017): 10^6^.

Figures 3, 5A, 6E, Tables 1–2.

Type locality: Myanmar: Myit Kyi Pauk Stream, Sittaung Basin [18.9613°N, 96.4455°E].

Holotype RMBH biv_251_3: Myanmar: Myit Kyi Pauk Stream, Sittaung Basin, 18.9613°N, 96.4455°E, 26 November 2016, Vikhrev leg.

Paratypes: type locality, 26.xi.2016, 2 specimens (RMBH nos. biv_251_1 and biv_251_2), Vikhrev leg.; Myanmar: Bilin River, near Yhin Ohn village, 17.3306°N, 97.2418°E, 13.ii.2018, 3 specimens (RMBH nos. biv371_1, biv371_2, and biv_371_3); Myanmar: Thae Phyu Stream, Bilin River, 17.2757°N, 97.1274°E, 14.ii.2018, 3 specimens (RMBH nos. biv375_1, biv375_2, and biv375_3); Myanmar: Moeyungyi Lake, 17.5968°N, 96.5950°E, 2 specimens (RMBH biv biv377_2 and biv377_3), 17.ii.2018; Myanmar: Bago River, 17.5334°N, 96.3315°E, 18.ii.2018, 2 specimens (RMBH nos. biv381_3 and biv381_4); Myanmar: Moe Lut Stream, Bago Basin, 17.6011°N, 96.2861°E, 18.ii.2018, 1 specimen (RMBH biv386_2), Bolotov, Vikhrev, Nyein Chan and local villagers leg.

Etymology: The name of this subspecies refers to its olive-green periostracum.

Diagnosis: This subspecies is conchologically similar to *Indochinella pugio pugio* (Benson, 1862), but differs from it by shorter and higher shell, more pronounced and curved lateral teeth, moderately strong sculpture on shell disc, and a diagnostic nucleotide substitution in the *COI* gene fragment (Table 2).

Description: Small mussel. Shell length 32.2–45.8 mm, height 16.3–23.8, width 11.5–16.9 mm. Shell cuneiform, inequilateral, rather inflated. Posterior ridge oblique. Shell sculpture moderately strong. Periostracum smooth, olive-green, nacre whitish. Umbo not very prominent, corrugated, beak sculpture little pronounced. Left valve with two short lateral teeth and two ribbed pseudocardinal teeth. Right valve with single short curved lateral tooth and two pseudocardinal teeth, anterior tooth not pronounced, posterior tooth strong, ribbed and triangular. Umbo cavity rather deep. Anterior adductor scar well pronounced, funneled; posterior adductor scar shallow.

Habitat and ecology: Sites with clay bottom substrate in various rivers, streams and lakes (Fig. 6E).

Distribution: Sittaung, Bilin, and Bago River basins, Myanmar.

***Indochinella pugio daweiensis*** **ssp. nov**.

= *Scabies crispata* sensu Bolotov *et al*. (2017): 6, Fig. 4^15^.

= *Oxynaia* sp. ‘Tavoy’ sensu Bolotov *et al*. (2017): 10^6^.

Figures 3, 5B, 6F, Tables 1–2.

Type locality: Myanmar: Dawei (Tavoy) River [14.5012°N, 98.1557°E].

Holotype RMBH biv_147_3: Myanmar: Dawei River, 14.5012°N, 98.1557°E, 26.iv.2015, Bolotov leg.

Paratypes: type locality, 26.iv.2015, 11 specimens (RMBH nos. biv147_10, biv147_18, biv148_4, biv148_7, biv148_15, biv147_30, biv147_1, biv147_2, biv147_4, biv148_2, and biv148_3), Bolotov leg.

Etymology: This local subspecies is named after Dawei River, in which the type series was collected.

Diagnosis: This taxon differs from the nominative subspecies in having oval-shaped shell, a more rounded posterior ridge and a more gradual posterior slope, distinct zigzag ridges across the shell disc (Fig. 5B), and fixed nucleotide substitutions in the *COI*, *16S rRNA* and *28S rRNA* gene fragments (Table 2). Perhaps, it is the most conchologically peculiar taxon within the genus.

Description: Very small mussel. Shell length 26.1–28.0 mm, height 11.5–20.4 mm, width 8.2–16.8 mm. Shell shape obovate, inequilateral, rather thick. Posterior ridge broader than the anterior ridge, oblique. Shell sculpture very strong, with distinct zigzag ridges across the shell disc. Periostracum sandy-brown with numerous dark-green zigzag ridges, nacre yellow-whitish. Umbo prominent, slightly corrugated, beak sculpture not very strong. Left valve with two short lateral teeth and two pseudocardinal teeth. Right valve with a single lateral tooth and blunt pseudocardinal tooth, anterior tooth not developed. Umbo cavity shallow and open. Anterior adductor scar marked, rounded or oval-shaped. Posterior adductor scar not pronounced, obovate.

Habitat and ecology: The subspecies is known only from its type series, which was collected from river sites with clay and gravel bottom substrate (Fig. 6F).

Distribution: Dawei River, southeastern Myanmar.

***Indochinella pugio paradoxa*** **ssp. nov**.

Figures 3, 5C, 6G, Tables 1–2.

Type locality: Myanmar: Haungthayaw River upstream of Kawkareik town [16.4714°N, 98.2182°E].

Holotype: RMBH biv361_1: Myanmar: Haungthayaw River upstream of Kawkareik town, 16.47144°N, 98.21825°E, 9.ii.2018, Nyein Chan leg.

Paratypes: type locality, 9.ii.2018, 8 specimens (RMBH nos. biv361_2, biv361_3, biv361_4, biv361_5, biv361_6, biv361_7, biv361_8, and biv361_9), Nyein Chan leg.

Etymology: The name of this subspecies refers to the paradox that it has morphological features similar to the *Scabies* group but is closely related genetically with *Indochinella pugio* group.

Diagnosis: The subspecies is very similar morphologically to representatives of the genus *Scabies* Haas, 1911, e.g. *S*. *crispata* (Gould, 1843), but it genetically sisters to the *Indochinella* clade. The new taxon can be distinguished from other *Indochinella pugio* subspecies by strong radial and w-shaped sculpture of the shell, biangular shape of the posterior end, and by fixed nucleotide substitutions in the *COI*, *16S rRNA* and *28S rRNA* gene fragments (Table 2).

Description: Small mussel. Shell length 22.3–36.1 mm, height 12.3–18.0 mm, width 8.2–13.1 mm. Shell somewhat cuneiform, inequilateral, rather thin, slightly inflated. Anterior end rounded, dorsal and ventral margins almost straight or slightly curved, parallel to each other at some specimens. Posterior end biangular, from umbo to upper angle covered by ridges. Umbo slightly elevated with w-shaped sculpture; umbo area well-separated. Periostracum green, concentrically striated, laminiferous, with dark-green zigzag ridges in lower half of the shell; nacre white-yellow. Lateral teeth lamellar, thin, straight or slightly curved, a single tooth on right valve and two teeth on left valve. Two pseudocardinal teeth on right valve, anterior tooth lamellar, posterior tooth trapezoidal; two pseudocardinal teeth on left valve, anterior tooth rectangular and ribbed, anterior tooth small and pyramidal. Anterior muscle attachment scar rounded, well-visible. Posterior muscle attachment scar shallow.

Habitat and ecology: This species is known from the same locality as *R. mouhoti haungthayawensis* **ssp. nov**. (Fig. 6G).

Distribution: Haungthayaw River, southeastern Myanmar.

### Subfamily Rectidentinae Modell, 1942

Type genus: *Rectidens* Simpson, 1900 (by original designation)

### Tribe Contradentini Modell, 1942

Type genus: *Contradens* Haas, 1911 (by original designation)

**Genus** ***Yaukthwa*** **Konopleva** ***et al*** *.* **, 2019**

Type species: *Yaukthwa nesemanni* (Konopleva, Vikhrev & Bolotov, 2017) (by original designation)

***Yaukthwa elongatula*** **sp. nov**.

Figures 3, 5D, 6H, Tables 1–2.

Type locality: Myanmar: Chindwin River [23.1918°N, 94.3217°E].

Holotype: RMBH biv346_2: Myanmar: Chindwin River, 23.1918°N, 94.3217°E, 2.ii.2018, Bolotov, Vikhrev, Lopes-Lima, Nyein Chan and local villagers leg.

Paratypes: type locality, 2.ii.2018, 2 specimens (RMBH nos. biv346_3 and biv346_1); Myanmar: Myit Tha (Manipur) River, 23.2006°N, 94.2214°E, 2.ii.2018, 3 specimens (RMBH nos. biv344_1, biv344_2, and biv344_3); Myanmar: Myit Tha (Manipur) River, 23.2448°N, 94.1661°E, 2.ii.2018, 2 specimens (biv341_2 and biv341_3); Myanmar: Myit Tha (Manipur) River, 23.2284°N, 94.1434°E, 2.ii.2018, 4 specimens (biv339_1, biv339_2, biv339_3, and biv339_7), Bolotov, Vikhrev, Lopes-Lima, Nyein Chan and local villagers leg.

Etymology: The name of this species refers to its elongated shell shape.

Diagnosis: The new species remotely resembles *Yaukthwa nesemanni*, but differs from it by much more elongated shell, broader posterior margin, narrower anterior margin, reduced pseudocardinal teeth, and fixed nucleotide substitutions in the *COI*, *16S rRNA* and *28S rRNA* gene fragments (Table 2).

Description: Medium-sized mussel. Shell length 35.1–64.3 mm, height 16.4–28.1 mm, width 8.2–14.9 mm. Shell trapezoidal, elongated, inequilateral, thin, not inflated. Anterior end rounded, very narrow at some specimens, dorsal side curved, ventral margin slightly concaved. Posterior end broader than anterior end, truncated. Umbo not projected, slightly elevated, strongly corrugated at some specimens; corrugation may cover almost entire shell. Periostracum from light- to dark-brown, nacre white-bluish with yellow regions. Lateral teeth very thin, slightly curved, a single tooth on right valve and two teeth on left valve. Pseudocardinal teeth reduced. Anterior muscle attachment scar oval or drop-like, visible. Posterior muscle attachment scar oval, shallow.

Habitat and ecology: Sites with hard clay and rocky bottom substrate in large, fast-flowing rivers (Fig. 6H).

Distribution: Myit Tha (Manipur) and Chindwin rivers, Ayeyarwady Basin, northwestern Myanmar.

## Discussion

### Taxonomic implications

Here, we introduce eight new mussel taxa belonging to the genera *Leoparreysia*, *Radiatula*, *Indochinella*, and *Yaukthwa*. *Leoparreysia* was established by us for a group of species from the Western Indochina Subregion that was previously placed within the Indian genus *Parreysia* because of their external similarity^6^. However, we found that the *Leoparreysia* members represent a separate phylogenetic clade of the Parreysiinae, which is distantly related to the true Parreysiini^6^. *Leoparreysia whitteni* **sp. nov**. appears to be the smallest representative of this group. This species was recorded from the Ayeyarwady Basin, which seems to be the primary evolutionary hotspot of Leoparreysiini diversity, with at least five valid species^6^.

*Radiatula* appears to be another endemic genus of Western Indochina^6,7^, while its sister groups, e.g. *Scabies* Haas, 1911, *Unionetta* Haas, 1955, and *Harmandia* Rochebrune, 1881, are distributed in the Sundaland Subregion^14^. In this study, we describe three additional members of *Radiatula*, i.e. *R. myitthanensis* **sp. nov**., *R. chindwinensis* **sp. nov**., and *R. mouhoti haungthayawensis* **ssp. nov**. Various *Radiatula* species are abundant throughout Myanmar^6^, while *R*. *crispisulcata*, the type species of this genus, is known only from old museum lots.

*Indochinella* was introduced by us for the *Oxynaia pugio* group as a monotypic genus, endemic to Western Indochina^7^. The *pugio*-group is characterized by a high genetic and morphological variability, indicating the presence of additional taxa^6,7^. In this study, we introduce three more subspecies-level lineages: *Indochinella pugio viridissima* **ssp. nov**. from the Sittaung, Bilin and Bago rivers, *I. pugio daweiensis* **ssp. nov**. from the Dawei River, and *I. pugio paradoxa* **ssp. nov**. from the Haungthayaw River (Fig. 2). The two latter subspecies differ from other taxa by one or two diagnostic substitutions in the nuclear *28S rRNA* gene fragment (Table 2). This feature is remarkable, because such differences in the slowly evolving nuclear genes for freshwater mussels often correspond to species-level divergence^16^. Furthermore, these two subspecies are conchologically more similar to the *Scabies* taxa than to the nominative subspecies of *I*. *pugio*.

*Yaukthwa* is a species-rich genus in the subfamily Rectidentinae^9^. Historically, all *Yaukthwa* taxa were placed within the genus *Trapezoideus* Simpson, 1900 that was thought to comprise several widespread species, e.g. *T. exolescens* (Gould, 1843)^17–19^. However, based on a multi-locus phylogeny, *Trapezoideus exolescens* was found to be a member of the Parreysiinae^13^, and it has subsequently been transferred to its own genus, *Trapezidens* Bolotov, Vikhrev & Konopleva, 2017^6^. A taxonomic revision of the tribe Contradentini revealed that the genus *Trapezoideus* is a monotypic taxon with a single species, *T. foliaceus*^9^. Several species from Western Indochina previously assigned to *Trapezoideus*^6^ were transferred to *Yaukthwa*^9^. This genus seems to be an endemic clade of Western Indochina, which includes a variety of endemic species with restricted distribution ranges, mostly in headwaters of rivers and streams^6,9^. *Yaukthwa elongatula* **sp. nov**. has a remarkable shell shape, which resembles the ultra-elongate representatives of the genus *Solenaia* (Gonideinae). This new species inhabits the Chindwin Basin, and it is associated with specific rocky and hard clay bottom sites.

While several recent integrative works^6,7,9,14,15,20–22^ have greatly improved our knowledge on the taxonomy and biogeography of freshwater mussels in Southeast Asia, many gaps in these fields are still to be filled. This novel study contributes to the further expansion and redefinition of the Oriental Unionidae. However, many nominal species of high importance for taxonomic and phylogenetic research, e.g. *Pseudodon crebristriatus* (Anthony, 1865) and *Modellnaia siamensis* Brandt, 1974, were not rediscovered by extensive field surveys during the last seven years^6,7,9,14,15^, and such taxa are in need of future research efforts.

### Patterns of endemism

Previously, we have considered every large or medium-sized freshwater basin in the Oriental Region to be a separate evolutionary hotspot of the Unionidae fauna^6,15^. Pfeiffer *et al*.^14^ noted that this point of view does not consider the intra-basin heterogeneity, when several evolutionary hotspots can be recorded within a single drainage basin. Additionally, it was suggested that the proportion of single-drainage endemics proposed by Bolotov *et al*.^15^ may have been overestimated that underestimated the role of inter-basin faunal exchanges in shaping distribution patterns of freshwater mussels in Southeast Asia^14^. Based on the newly obtained results, we partly agree with those comments and propose an updated classification scheme for endemic freshwater mussel taxa from Southeast Asia as follows:

(1) Subregion endemics: widespread taxa, the range of which encompasses several freshwater drainages within a single biogeographic subregion. This group seems to contain a rather small number of taxa from Western Indochina, e.g. *Lamellidens savadiensis* (Nevill, 1877), *Indochinella pugio viridissima* **ssp. nov**., and *Leoparreysia tavoyensis* (Gould, 1843). A few examples of species with rather broad ranges are known from the Sundaland Subregion, e.g. *Scabies crispata* (Gould, 1843), *S. phaselus* (Lea, 1856), and *S. mandarinus* (Morelet, 1864)^14^. Several freshwater mussel species are widespread in rivers of the Malay Peninsula and northern Borneo^20,21^. Such multi-drainage distribution patterns were likely originated by dispersal events via direct connections between freshwater basins during the Late Pleistocene^6,14,15^.

(2) Single-drainage endemics. At first glance, the majority of freshwater mussels from Western Indochina may belong to this group, e.g. *Leoparreysia whitteni* **sp. nov**., *L. burmana* (Blanford, 1869), *Radiatula mouhoti haungthayawensis* **ssp. nov**., *Indochinella pugio daweiensis* **ssp. nov**., and *I. pugio paradoxa* **ssp. nov**.

(3) Intra-drainage endemics: taxa with local ranges corresponding to a certain section within a freshwater basin. Currently, members of this group from Western Indochina are poorly known, because our previous field surveys were primarily focused on sampling taxa from separate freshwater drainages^6,15^. Currently, a few species can be assigned to this group, e.g. *Radiatula myitthanensis* **sp. nov**., *R. chindwinensis* **sp. nov**., *Yaukthwa elongatula* **sp. nov**., *Y. inlenensis* Konopleva *et al*., 2019, and *Y. paiensis* Konopleva *et al*.^9^. However, a number of local endemics from other sites may be overlooked.

## Methods

### Data sampling

Samples of freshwater mussels were collected by hand from different water bodies throughout Myanmar using a rapid bioassessment approach of Cummings *et al*.^23^. A series of each mussel morphospecies from every study site has been collected. Soft tissue snips for DNA analyses were preserved in 96% ethanol immediately after collection.

### Molecular data and phylogenetic analyses

New *COI*, *16S rRNA* and *28S rRNA* gene sequences were generated from 52 freshwater mussel specimens using a standard approach following published works^6,9,15^. The sequence data set and outgroup taxa are presented in Supplementary Table 1. The sequence alignment of each gene fragments was performed separately using the Muscle algorithm of MEGA7^24^. The aligned data sets were concatenated into a multi-locus alignment. Absent sites were coded as missing data. Five partitions, i.e. three codons of *COI* + *16S rRNA* + *28S rRNA*, were used for phylogenetic analysis. We carried out maximum likelihood phylogenetic searches using web interface and server for IQ-TREE (W-IQ-TREE)^25–27^ with an automatic identification of the best-fit substitution model for each partition (Supplementary Table 2). To estimate the probability of internal branches, an ultrafast bootstrap (UFBoot) algorithm^28^ with 10,000 replicates was applied. Bayesian models were implemented in MrBayes v. 3.2.6^29^ with two runs, each with three heated (temperature = 0.1) and one cold Markov chain (30,000,000 generations with sampling every 1000th generation) at the San Diego Supercomputer Center through the CIPRES Science Gateway^30^. The first 15% of trees were discarded as burn-in. Tracer v. 1.6^31^ was used to check a convergence of the MCMC chains to a stationary distribution.

### Species delimitation and diagnostics of new taxa

For preliminary delimitation of the putative species-level clades, we used BEAST2 v.2.5.1^32^ with STACEY v.1.2.4^33^ package. Each sequence in the multi-locus alignment (see above) was initially assigned to a prospective species unit. *Nitia teretiuscula* and *Coelatura* aff. *aegyptiaca* were used as outgroup (Supplementary Table 1). Five partitions (three codons of *COI* + *16S rRNA* + *28S rRNA*) were analyzed under a HKY substitution model and lognormal relaxed clock algorithm. The priors for the Birth Death Model were applied as follows: CollapseHeight = 0.001, Relative Death rate = 0.5, and Relative Collapse Weight = 0.5 using a beta prior. All parameters were estimated. Each run was conducted for 100,000,000 generations with sampling every 5000th tree and 10% burn-in. The calculations were performed at the San Diego Supercomputer Center through the CIPRES Science Gateway^30^. A matrix of putative species-level clusters based on SMC-trees generated by STACEY was constructed using SpeciesDelimitationAnalyser with initial 10% burn-in (www.indriid.com/software.html). An uncorrected *COI* mean *p*-distance to the nearest neighbor of each lineage was calculated in MEGA7^24^. To check the putative subspecies-level units having a rather low molecular distance from nearest neighbors, we additionally used a network-based approach using Network v. 4.6.1.3 software with default settings^34^ that can reveal less prominent genetic differences between prospective taxa.

After a phylogenetic species delimitation, we estimated morphological differences between a new taxon and closely related (congeneric) taxa. The comparative analysis of the shell morphology was carried out with a special focus to the shell shape, structure of pseudocardinal and lateral teeth, shape of muscle attachment scars, and umbo position^6,7,9,13^. Three shell dimensions of each specimen, included in the type series of new taxa, i.e., the length, height, and width of the shell (all at the maximum diameter), were measured using calipers (±0.1 mm).

Finally, the molecular diagnosis of every new taxon was designed using fixed nucleotide substitutions, which were estimated for each gene separately using a Toggle Conserved Sites tool of MEGA7^24^ at 50% level. For each new taxon, an alignment of congeneric haplotype sequences was performed using the Muscle algorithm implemented in MEGA7^24^. All deleterious mutations were retained for the analyses.

### Nomenclatural acts

The electronic edition of this article conforms to the requirements of the amended International Code of Zoological Nomenclature (ICZN), and hence the new names contained herein are available under that Code from the electronic edition of this article. This published work and the nomenclatural acts it contains have been registered in ZooBank (http://zoobank.org), the online registration system for the ICZN. The LSID for this publication is: urn:lsid:zoobank.org:pub:B017A518-0FBE-40F5-A2A4-8C13AC7E80BC. The electronic edition of this paper was published in a journal with an ISSN, and has been archived and is available from PubMed Central.

## Supplementary information

Supplementary Info

## Data Availability

The type series of the new species are available in the RMBH – Russian Museum of Biodiversity Hotspots, Federal Center for Integrated Arctic Research, Russian Academy of Sciences, Arkhangelsk, Russia. The sequences generated in this study are available from GenBank. GenBank accession number and collecting locality for each specimen are presented in Supplementary Table 1.
