# Supplementary material for: Eight new freshwater mussels (Unionidae) from tropical Asia
Source: Sci Rep. 2019 Aug 19;9:12053. doi: 10.1038/s41598-019-48528-z (PMC6700347; doi:10.1038/s41598-019-48528-z)
Supplement: Supplementary file 1 — Supplementary Info [file 41598_2019_48528_MOESM1_ESM.pdf]

SUPPLEMENTARY INFORMATION

Eight new freshwater mussels (Unionidae) from tropical Asia

Ivan N. Bolotov, Ekaterina S. Konopleva, Ilya V. Vikhrev, Manuel Lopes-Lima, Arthur E. Bogan, Zau Lunn, Nyein Chan, Than Win, Olga V. Aksenova, Mikhail Yu. Gofarov, Alena A. Tomilova & Alexander V. Kondakov

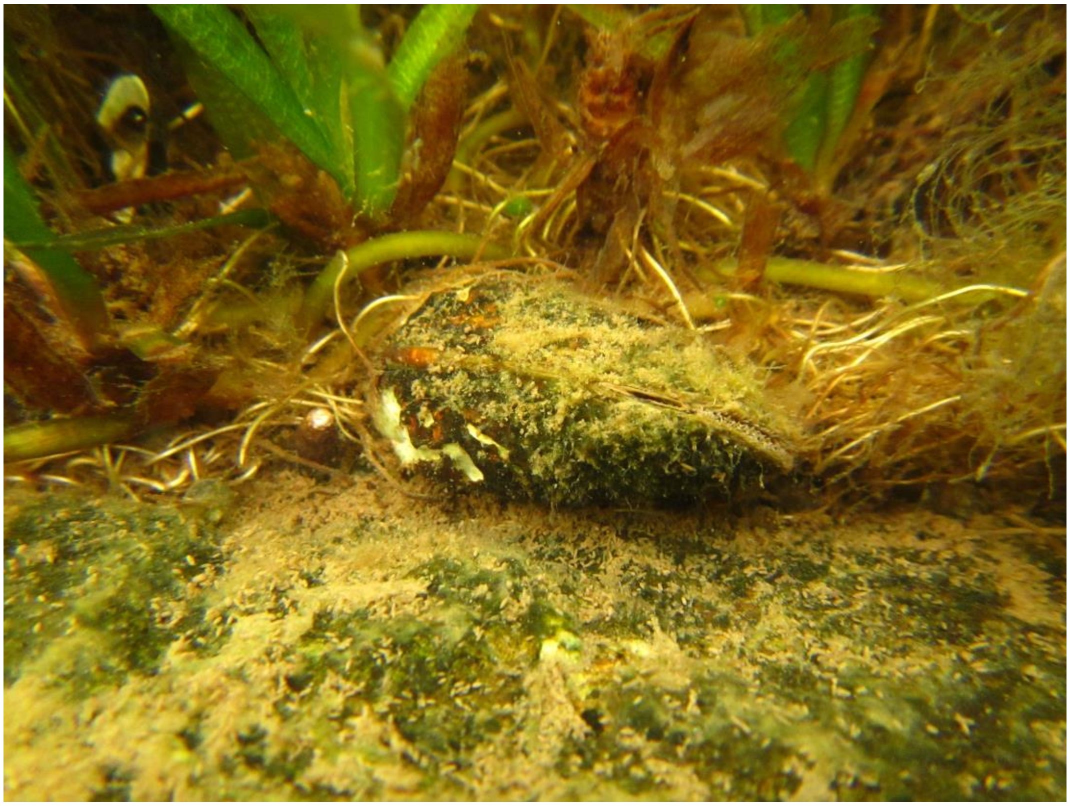

Radiatula myitkyinae (Prashad, 1930) on basalt outcrop in Lake Indawgyi (Photo: Ivan N. Bolotov)

Supplementary Table 1. List of sequences used in this study, including the species, locality and NCBI's GenBank accession numbers

Species	Locality	Specimen Voucher	*COI*	*16S rRNA*	*28S rRNA*
**UNIONIDAE Rafinesque, 1820**					
**PARREYSIINAE Henderson 1935**					
**Indochinellini Bolotov, Pfeifer, Vikhrev & Konopleva, 2018**					
Indochinella pugio pugio (Benson, 1862)	Myanmar: Ayeyarwady River basin, Lake Nant Phar	RMBH biv_258_1	MF352261	MF352325	MF352386
I. pugio pugio (Benson, 1862)	Myanmar: Ayeyarwady River basin, Lake Nant Phar	RMBH biv_258_2	MF352262	MF352326	MF352389
I. pugio pugio (Benson, 1862)	Myanmar: Ayeyarwady River basin, Lake Myaung	RMBH biv_268_1	MF352285	n/a	n/a
I. pugio pugio (Benson, 1862)	Myanmar: Ayeyarwady River basin, Lake Myaung	RMBH biv_268_2	MF352286	MF352346	MF352403
I. pugio pugio (Benson, 1862)	Myanmar: Ayeyarwady River basin, Lake Myaung	RMBH biv_268_4	MF352287	MF352347	MF352404
I. pugio pugio (Benson, 1862)	Myanmar: Ayeyarwady River basin, Chindwin River, Pauk In Lake	UA 20739	JN243899	KP795046	JN243879
I. pugio viridissima **ssp. nov.**	Myanmar: Sittaung River basin, Myit Kyi Pauk stream	RMBH biv_ 251_1	MF352242	MF352312	MF352370
I. pugio viridissima **ssp. nov.**	Myanmar: Sittaung River basin, Myit Kyi Pauk stream	RMBH biv _251_2	MF352243	MF352313	MF352371
I. pugio viridissima **ssp. nov.**	Myanmar: Sittaung River basin, Myit Kyi Pauk stream	RMBH biv_251_3	MF352244	MF352314	MF352372
I. pugio viridissima **ssp. nov.**	Myanmar: Bilin River	RMBH biv_371_3	MK372428	n/a	n/a
I. pugio viridissima **ssp. nov.**	Myanmar: Bilin River	RMBH biv_375_1	MK372429	n/a	n/a
I. pugio viridissima **ssp. nov.**	Myanmar: Bilin River	RMBH biv_375_2	MK372430	n/a	n/a
I. pugio viridissima **ssp. nov.**	Myanmar: Moeyungyi Lake, Sittaung Basin	RMBH biv_377_2	MK372432	n/a	n/a
I. pugio viridissima **ssp. nov.**	Myanmar: Moeyungyi Lake, Sittaung Basin	RMBH biv_377_3	MK372433	n/a	n/a
I. pugio viridissima **ssp. nov.**	Myanmar: Bago River	RMBH biv_381_3	MK372434	n/a	n/a
I. pugio viridissima **ssp. nov.**	Myanmar: Bago River	RMBH biv_381_4	MK372435	n/a	n/a

Species	Locality	Specimen Voucher	*COI*	*16S rRNA*	*28S rRNA*
I. pugio viridissima **ssp. nov.**	Myanmar: Moe Lut Stream, Bago Basin	RMBH biv_386_2	MK372436	n/a	n/a
I. pugio daweiensis **ssp. nov.**	Myanmar: Dawei River	RMBH biv_147_3	KX865852	KX865623	KX865724
I. pugio daweiensis **ssp. nov.**	Myanmar: Dawei River	RMBH biv_147_10	KX865853	KX865624	KX865725
I. pugio daweiensis **ssp. nov.**	Myanmar: Dawei River	RMBH biv_147_18	KX865854	KX865625	KX865726
I. pugio daweiensis **ssp. nov.**	Myanmar: Dawei River	RMBH biv_148_4	KX865855	KX865626	KX865727
I. pugio daweiensis **ssp. nov.**	Myanmar: Dawei River	RMBH biv_148_7	KX865856	KX865627	KX865728
I. pugio daweiensis **ssp. nov.**	Myanmar: Dawei River	RMBH biv_148_15	KX865857	KX865628	KX865729
I. pugio daweiensis **ssp. nov.**	Myanmar: Dawei River	RMBH biv_147_30	MK372395	n/a	n/a
I. pugio paradoxa **ssp. nov.**	Myanmar: Haungthayaw River	RMBH biv_361_1	MK372420	MK372465	MK372496
I. pugio paradoxa **ssp. nov.**	Myanmar: Haungthayaw River	RMBH biv_361_2	MK372421	MK372466	MK372497
I. pugio paradoxa **ssp. nov.**	Myanmar: Haungthayaw River	RMBH biv_361_3	MK372422	MK372467	MK372498
Radiatula mouhoti Vikhrev, Bolotov & Konopleva, 2017	Myanmar: Sittaung River near Taungoo	RMBH biv_248_1	MF352234	MF352305	MF352363
R. mouhoti Vikhrev, Bolotov & Konopleva, 2017	Myanmar: Sittaung River near Taungoo	RMBH biv_248_4	MF352236	MF352306	MF352364
R. mouhoti Vikhrev, Bolotov & Konopleva, 2017	Myanmar: Sittaung River near Taungoo	RMBH biv_253_1	MF352248	MF352317	MF352375
R. mouhoti haungthayawensis **ssp. nov.**	Myanmar: Haungthayaw River	RMBH biv_360_1	MK372417	MK372463	MK372493
R. mouhoti haungthayawensis **ssp. nov.**	Myanmar: Haungthayaw River	RMBH biv_360_2	MK372418	MK372464	MK372494
R. mouhoti haungthayawensis **ssp. nov.**	Myanmar: Haungthayaw River	RMBH biv_360_3	MK372419	n/a	MK372495
R. chaudhurii (Preston, 1912) [=*R.* cf. *bonneaudii* sp.1 sensu Bolotov et al.,	Myanmar: Ayeyarwady River basin, Tar Pein River	RMBH biv_260_5	MF352266	MF352330	MF352390

Species	Locality	Specimen Voucher	*COI*	*16S rRNA*	*28S rRNA*
2017]					
R. chaudhurii (Preston, 1912) [=*R.* cf. *bonneaudii* sp.1 sensu Bolotov et al., 2017]	Myanmar: Ayeyarwady River basin, Tar Pein River	RMBH biv_260_10	MF352268	MF352332	MF352392
R. chaudhurii (Preston, 1912) [=*R.* cf. *bonneaudii* sp.1 sensu Bolotov et al., 2017]	Myanmar: Ayeyarwady River basin, Tar Pein River	RMBH biv_260_9	MF352267	MF352331	MF352391
R. chaudhurii (Preston, 1912) [=*R.* cf. *bonneaudii* sp.1 sensu Bolotov et al., 2017]	Myanmar: Ayeyarwady River basin, midstream near Tha Phan Kone village	RMBH biv_427_2	MK372445	n/a	n/a
R. chaudhurii (Preston, 1912) [=*R.* cf. *bonneaudii* sp.1 sensu Bolotov et al., 2017]	Myanmar: Ayeyarwady River basin, midstream near Tha Phan Kone village	RMBH biv_427_1	MK372444	n/a	n/a
R. chaudhurii (Preston, 1912) [=*R.* cf. *bonneaudii* sp.1 sensu Bolotov et al., 2017]	Myanmar: Ayeyarwady River basin, midstream near Thin Baw Kone village	RMBH biv_421_3	MK372440	MK372472	MK372504
R. chaudhurii (Preston, 1912) [=*R.* cf. *bonneaudii* sp.1 sensu Bolotov et al., 2017]	Myanmar: Ayeyarwady River basin, midstream near Thin Baw Kone village	RMBH biv_421_2	MK372439	MK372471	MK372503
R. myitkyinae (Prashad, 1930)	Myanmar: Ayeyarwady River basin, Lake Indawgyi	RMBH biv_107_1	KX865838	KX865609	KX865710
R. myitkyinae (Prashad, 1930)	Myanmar: Ayeyarwady River basin, Lake Indawgyi	RMBH biv_106_3	KX865842	KX865614	KX865714
R. myitkyinae (Prashad, 1930)	Myanmar: Ayeyarwady River basin, Lake Indawgyi	RMBH biv_106_2	KX865843	KX865613	KX865715
R. chindwinensis **sp. nov.**	Myanmar: Myit Tha (Manipur) River	RMBH biv_345_2	MK372405	MK372453	MK372483
R. chindwinensis **sp. nov.**	Myanmar: Chindwin River	RMBH biv_348_2	MK372410	MK372458	MK372488
R. chindwinensis **sp. nov.**	Myanmar: Myit Tha (Manipur) River	RMBH biv_345_1	MK372404	MK372452	MK372482
R. chindwinensis **sp. nov.**	Myanmar: Nay Chin Sayar River	RMBH biv_357_1	MK372414	MK372462	MK372492
R. chindwinensis **sp. nov.**	Myanmar: Nay Chin Sayar River	RMBH biv_357_2	MK372415	n/a	n/a
R. chindwinensis **sp. nov.**	Myanmar: Nay Chin Sayar River	RMBH biv_357_3	MK372416	n/a	n/a
R. myitthanensis **sp. nov.**	Myanmar: Myit Tha	RMBH	MK372396	MK372450	MK372477

Species	Locality	Specimen Voucher	*COI*	*16S rRNA*	*28S rRNA*
	(Manipur) River	biv_337_3			
R. myitthanensis **sp. nov.**	Myanmar: Chindwin River	RMBH biv_348_1	MK372409	MK372457	MK372487
**Leoparreysiini Vikhrev, Bolotov & Kondakov, 2017**					
Leoparreysia olivacea (Prashad, 1930)	Myanmar: Ayeyarwady River, 3 mi SW Pakkoku, Magway Division. 21.2963° N, 95.0198° E	UMMZ 304641	KP795022	KP795044	KP795005
L. canefrii Vikhrev, Bolotov & Kondakov, 2017	Myanmar: Sittaung River near Taungoo	RMBH biv_249	MF352237	MF352307	MF352365
L. canefrii Vikhrev, Bolotov & Kondakov, 2017	Myanmar: Sittaung River near Taungoo	RMBH biv_252_1	MF352245	MF352315	MF352373
L. canefrii Vikhrev, Bolotov & Kondakov, 2017	Myanmar: Sittaung River near Taungoo	RMBH biv_252_2	MF352246	MF352316	MF352374
L. burmana (Blanford, 1869)	Myanmar: Ayeyarwady River	RMBH biv_424_3	MK372443	MK372474	MK372506
L. burmana (Blanford, 1869)	Myanmar: Ayeyarwady River	RMBH biv_443_3	MK372447	n/a	n/a
L. burmana (Blanford, 1869)	Myanmar: Chindwin River	RMBH biv_350_2	MK372413	MK372461	MK372491
L. burmana (Blanford, 1869)	Myanmar: Ayeyarwady River	RMBH biv_424_2	MK372442	MK372473	MK372505
L. burmana (Blanford, 1869)	Myanmar: Ayeyarwady River	RMBH biv_445_1	MK372448	n/a	n/a
L. burmana (Blanford, 1869)	Myanmar: Ayeyarwady River	RMBH biv_419_3	MK372438	MK372470	MK372502
L. burmana (Blanford, 1869)	Myanmar: Chindwin River	RMBH biv_350_1	MK372412	MK372460	MK372490
L. burmana (Blanford, 1869)	Myanmar: Ayeyarwady River	RMBH biv_419_2	MK372437	n/a	n/a
L. burmana (Blanford, 1869)	Myanmar: Ayeyarwady River	RMBH biv_424_1	MK372441	n/a	n/a
L. tavoyensis (Gould, 1843)	Myanmar: Dawei River	RMBH biv_149	KX230543	KX230554	KX230565
L. tavoyensis (Gould, 1843)	Myanmar: Haungthayaw River	RMBH biv_362_1	MK372423	n/a	MK372499
L. tavoyensis (Gould, 1843)	Myanmar: Haungthayaw River	RMBH biv_362_2	MK372424	n/a	n/a
L. tavoyensis (Gould, 1843)	Myanmar: Haungthayaw River	RMBH biv_362_3	MK372425	n/a	n/a
L. whitteni **sp. nov.**	Myanmar: Chindwin River	RMBH	MK372411	MK372459	MK372489

Species	Locality	Specimen Voucher	*COI*	*16S rRNA*	*28S rRNA*
		biv_349			
L. whitteni **sp. nov.**	Myanmar: Ayeyarwady River	RMBH biv_435	MK372446	MK372475	MK372507
L. whitteni **sp. nov.**	Myanmar: Ayeyarwady River	RMBH biv_449	MK372449	MK372476	MK372508
**Coelaturini Modell, 1942**					
Nitia teretiuscula (Philippi, 1847)	Egypt	ANSP 416305	JN243897	n/a	JN243875
Coelatura aff. *aegyptiaca* (Cailliaud, 1827)	Egypt	ANSP 416304	JN243894	KP795045	JN243872
**RECTIDENTINAE Modell, 1942**					
**Contradentini Modell, 1942**					
Yaukthwa nesemanni (Konopleva, Vikhrev & Bolotov, 2017)	Myanmar: Sittaung River basin, Thauk Ye Kupt River	RMBH biv_144_14	KX865906	KX865663	KX865777
Y. nesemanni (Konopleva, Vikhrev & Bolotov, 2017)	Myanmar: Sittaung River basin, Thauk Ye Kupt River	RMBH biv_144_25	KX865907	KX865664	KX865778
Y. nesemanni (Konopleva, Vikhrev & Bolotov, 2017)	Myanmar: Sittaung River basin, Thauk Ye Kupt River	RMBH biv_144_19	KX865908	KX865665	KX865779
Y. panhai (Konopleva, Bolotov & Kondakov, 2017)	Myanmar: Sittaung River basin, Kyan Hone River	RMBH biv_138_4	KX865909	KX865666	KX865780
Y. panhai (Konopleva, Bolotov & Kondakov, 2017)	Myanmar: Sittaung River basin, Kyan Hone River	RMBH biv_138_12	KX865913	KX865670	KX865784
Y. panhai (Konopleva, Bolotov & Kondakov, 2017)	Myanmar: Sittaung River basin, Kyan Hone River	RMBH biv_155_4	KX865911	KX865668	KX865782
Y. inlenensis Konopleva et al., 2019	Myanmar: Salween River basin, Inle Lake Channel	RMBH biv_114_1	KX865915	KX865672	KX865786
Y. inlenensis Konopleva et al., 2019	Myanmar: Salween River basin, Inle Lake Channel	RMBH biv_114_2	KX865917	KX865674	KX865788
Y. inlenensis Konopleva et al., 2019	Myanmar: Salween River basin, Mway Stream	RMBH biv_139_7	KX865922	KX865676	KX865793
Y. cf. *dalliana* (Frierson, 1913)	Myanmar: Ayeyarwady River basin, Nanyinhka Chaung River	RMBH biv_111_21	KX865890	KX865657	KX865761
Y. cf. *dalliana* (Frierson, 1913)	Myanmar: Ayeyarwady River basin, Mali Hka River basin, Pan Khai stream	RMBH biv_101_5	KX865894	KX865659	KX865763
Y. cf. *dalliana* (Frierson,	Myanmar: Ayeyarwady River basin, Mali Hka River	RMBH	KX865899	KX865660	KX865768

Species	Locality	Specimen Voucher	*COI*	*16S rRNA*	*28S rRNA*
1913)	basin, Nam Palat River	biv_102_7			
Y. elongatula **sp. nov.**	Myanmar: Chindwin River	RMBH biv_346_2	MK372407	MK372455	MK372485
Y. elongatula **sp. nov.**	Myanmar: Chindwin River	RMBH biv_346_3	MK372408	MK372456	MK372486
Y. elongatula **sp. nov.**	Myanmar: Chindwin River	RMBH biv_346_1	MK372406	MK372454	MK372484
Y. elongatula **sp. nov.**	Myanmar: Myit Tha (Manipur) River	RMBH biv_344_3	MK372403	n/a	n/a
Y. elongatula **sp. nov.**	Myanmar: Myit Tha (Manipur) River	RMBH biv_344_1	MK372402	n/a	n/a
Y. elongatula **sp. nov.**	Myanmar: Myit Tha (Manipur) River	RMBH biv_341_3	MK372401	n/a	MK372481
Y. elongatula **sp. nov.**	Myanmar: Myit Tha (Manipur) River	RMBH biv_339_3	MK372399	n/a	n/a
Y. elongatula **sp. nov.**	Myanmar: Myit Tha (Manipur) River	RMBH biv_339_1	MK372397	n/a	MK372478
Y. elongatula **sp. nov.**	Myanmar: Myit Tha (Manipur) River	RMBH biv_339_2	MK372398	n/a	MK372479
Y. elongatula **sp. nov.**	Myanmar: Myit Tha (Manipur) River	RMBH biv_341_2	MK372400	MK372451	MK372480
**Outgroup Taxa**					
**MARGARITIFERIDAE Henderson, 1929**					
Gibbosula laosensis (Lea, 1863)	Laos: Mekong River basin, Nam Long River	RMBH biv 186_1	JX497731	KC845943	KT343741
Margaritifera dahurica (Middendorff,1850)	Far East of Russia: Amur River basin, Ilistaya River	RMBH biv 92_6	KJ161516	KJ943526	KT343747
M. margaritifera (Linnaeus, 1758)	Northwestern Russia: Onega River basin, Somba River	RMBH biv 618	KX550089	KX550091	KX550093
M. laevis (Haas, 1910)	Far East of Russia: Kurile Archipelago, Kunashir Island, Sennaya River	RMBH biv d0036/22	KJ161500	KJ943523	KT343742
M. middendorffi (Rosén, 1926)	Far East of Russia: Kamchatka, Bolshaya River basin, Nachilova River	RMBH biv d0099/6	KJ161547	KJ943528	KT343745
**IRIDINIDAE Swainson, 1840**					
Aspatharia pfeifferiana (Bernardi, 1860)	Zambia: Chambeshi River	BIVAToL-330	KC429107	KC429264	n/a
Chambardia wahlbergi (Krauss, 1848)	Zambia: Zambezi River	ANSP 419403	JN243886	KP184845	JN243864

Species	Locality	Specimen Voucher	*COI*	*16S rRNA*	*28S rRNA*
**ETHERIIDAE Deshayes, 1832**					
Etheria elliptica Lamarck, 1807	Zambia: Chambeshi River	FMNH 343390	KP184897	KP184847	KP184873
**MYCETOPODIDAE Gray, 1840**					
Anodontites elongata (Swainson, 1823)	Peru	FMNH 343931	KP184896	KP184846	KP184872
**HYRIIDAE Swainson, 1840**					
Triplodon corrugatus (Lamarck, 1819)	Peru	ANSP 416338	JN243890	KP184851	JN243868
Castalia ambigua Lamarck, 1819	Peru	ANSP 416341	JN243889	KP184848	JN243867
Microdontia anodontaeformis (Tapparone Canefri, 1883)	Guyana	UMMZ 304509	KP184909	KP184861	KP184885
Alathyria jacksoni Iredale, 1934	New Guinea	UMMZ 304512	KP184912	KP184864	KP184888
A. pertexta Iredale, 1934	Australia: New South Wales	UMMZ 304510	KP184910	KP184862	KP184886
A. profuga (Gould, 1850)	Australia: Queensland	UMMZ 304513	KP184913	KP184865	KP184889
Lortiella froggatti Iredale, 1934*	Australia: New South Wales	n/a	AF231746	KP184867	KP184891
Velesunio ambiguus (Philippi, 1847)	Western Australia	FMNH 337195	KP184915	KP184868	KP184892
**TRIGONIIDAE Lamarck, 1819**					
Neotrigonia margaritacea (Lamarck, 1804)*	Tasmania and Australia	n/a	U56850	DQ280034	DQ279963
N. lamarckii (Gray, 1838)	Australia: Coral Sea, North Stradbroke Island, Queensland	BIVAToL-97	KC429105	KC429262	KC429443

*Chimeric sequences.

Supplementary Table 2. Models of sequence evolution for each partition based on Bayesian information criterion (BIC) of jModelTest 2[1,2] and IQ-TREE[3,4]

Partition	Model by jModelTest 2	Model by IQ-TREE
COI		
1st codon of *COI*	TPM1uf	K3Pu+G
2nd codon of *COI*	TPM3uf+G	TPM3u+G
3rd codon of *COI*	TrN+I+G	TN+I+G
28S rRNA	TIM3+I+G	TIM3+I+G
16S rRNA	TIM2+G	TIM2+I+G

Supplementary References

1. Darriba, D., Taboada, G. L., Doallo, R. & Posada, D. jModelTest 2: more models, new heuristics and parallel computing. *Nature Methods* **9**, 772; DOI: 10.1038/nmeth.2109 (2012).
2. Guindon, S. & Gascuel, O. A simple, fast and accurate method to estimate large phylogenies by maximum-likelihood. *Systematic Biology* **52**, 696–704; DOI: 10.1080/10635150390235520 (2003).
3. Trifinopoulos, J., Nguyen, L. T., von Haeseler, A. & Minh, B. Q. W-IQ-TREE: a fast online phylogenetic tool for maximum likelihood analysis. *Nucleic Acids Research* **44**, W232–W235; DOI: 10.1093/nar/gkw256 (2016).
4. Kalyaanamoorthy, S., Minh, B. Q., Wong, T.K.F, von Haeseler, A. & Jermiin, L. S. ModelFinder: Fast model selection for accurate phylogenetic estimates. *Nature Methods* **14**, 587–589; DOI: 10.1038/nmeth.4285 (2017).
